# National weighting of data from the Behavioral Risk Factor Surveillance System (BRFSS)

**DOI:** 10.1186/s12874-016-0255-7

**Published:** 2016-11-15

**Authors:** Ronaldo Iachan, Carol Pierannunzi, Kristie Healey, Kurt J. Greenlund, Machell Town

**Affiliations:** 1 ICF International, 530 Gaither Road, Rockville, MD 20850 USA; 2 National Center for Chronic Disease Prevention and Health Promotion, Centers for Disease Control and Prevention, 4770 Buford Highway, N.E, Atlanta, GA 30341 USA

**Keywords:** BRFSS, Survey sampling, Weighting

## Abstract

**Background:**

The Behavioral Risk Factor Surveillance System (BRFSS) is a network of health-related telephone surveys--conducted by all 50 states, the District of Columbia, and participating US territories—that receive technical assistance from CDC. Data users often aggregate BRFSS state samples for national estimates without accounting for state-level sampling, a practice that could introduce bias because the weighted distributions of the state samples do not always adhere to national demographic distributions.

**Methods:**

This article examines six methods of reweighting, which are then compared with key health indicator estimates from the National Health Interview Survey (NHIS) based on 2013 data.

**Results:**

Compared to the usual stacking approach, all of the six new methods reduce the variance of weights and design effect at the national level, and some also reduce the estimated bias. This article also provides a comparison of the methods based on the variances induced by unequal weighting as well as the bias reduction induced by raking at the national level, and recommends a preferred method.

**Conclusions:**

The new method leads to weighted distributions that more accurately reproduce national demographic characteristics. While the empirical results for key estimates were limited to a few health indicators, they also suggest reduction in potential bias and mean squared error. To the extent that survey outcomes are associated with these demographic characteristics, matching the national distributions will reduce bias in estimates of these outcomes at the national level.

## Background

The Behavioral Risk Factor Surveillance System (BRFSS) is a network of health-related telephone surveys—conducted by all 50 states, the District of Columbia, and participating US territories—that receive technical assistance from CDC [1]. Annually, in the national aggregate, the BRFSS exceeds 400,000 interviews, with questions focusing on health-related risk behaviors, chronic health conditions, and use of preventive services. Each state samples from adults (aged 18 and older) living in private residences using an overlapping, dual frame landline and cell phone sample.

The BRFSS includes a core standardized questionnaire with optional modules of set questions that states may adopt according to their needs [1]. CDC provides guidance to data users on the appropriate weights to use if variables in analyses are taken from modules used by some of the states or taken from split samples. BRFSS data users often aggregate the state samples from the core questionnaire to use as a national database—without accounting for the state-level sampling of the data. Currently, CDC provides no additional guidance to BRFSS data users on how to adjust the weights provided for each individual state sample when they try to aggregate the state samples. As a result, these data users could introduce bias because the weighted distributions of the state samples do not always adhere to national demographic distributions. This article describes the statistical methodology we developed to compute national weights, as well as weighted national estimates and variance estimates, using BRFSS data aggregated across states.

The BRFSS currently uses a fully overlapping sample of landline and cell phone numbers. Currently, states must complete 35% of all interviews by cell phone, although some states interview as much as 65% of their samples by cell phone. States adopt a standard calling protocol each year [1]. States determine a sample design by constructing one or more sub-state regions from which strata will be taken. Given the ability to determine location from landline phone numbers, allocation of landline numbers to strata is a relatively straightforward process. Landline samples also adopt an additional stratification. In this method, known as disproportionate stratified sampling or DSS, telephone numbers are classified into areas of high or medium residential strata. Numbers are taken from the strata at a ratio of 1.5:1, respectively, in order to increase sample efficiency. Landline interviews also include within-household sampling, since phones are generally shared among adults within the home.

Locations for cell phone numbers are more difficult to pinpoint. Some information on geostrata can be obtained from samples drawn from rate centers or billing information. In other cases, locational information is derived from respondents themselves, when asked about county and zip code. If a person has moved from one state to another and retained a cell phone number, the respondent is interviewed and data are then transferred to the state where the respondent actually resides. A cell phone respondent with a Georgia phone number prefix who actually lives in Tennessee, might therefore be interviewed by Georgia but have his/her data transferred to Tennessee after the interview was completed [1].

Once data are collected, CDC provides technical assistance to the states by weighting the data with a method called raking. The margins used for raking are the same for each state, although categories may be collapsed differently for some margins in different states. Weighting variables include age, race, sex, education, ethnicity, marital status, home ownership, sub-state region, and phone ownership (landline only, cell phone only, or dual user). CDC also assists states with data cleaning and data-quality reporting and releases a public-use data set. In 2011, the BRFSS moved from a simpler post-stratification process to raking [2] and strengthened its standardized protocols to allow for the inclusion of cell-phone interviews.

Users may take national estimates of health-related outcomes from a number of national health-data sources, such as the National Health Interview Survey (NHIS), the National Health and Nutrition Examination Survey (NHANES), and the National Survey on Drug Use and Health (NSDUH)—all of which provide estimates on topics also found in the BRFSS. State-level estimates of BRFSS are useful for many different types of research, but many data users also need to generate national estimates from BRFSS--which often is the only provider (or one of a limited number of providers) of health indicator data, or with a much larger number of respondents than other surveys (see Table 1). For example, the NHIS includes a number of items on food security including skipping meals, concern about having enough food, and not eating balanced meals [3], while the BRFSS includes specific items on what individual respondents have eaten [1] with a large enough sample to provide information that can be broken down by demographic subgroups. For these and other reasons, researchers might select the BRFSS when producing national estimates. Example prevalence estimates that have been published based on BRFSS data aggregated nationally include estimates for conditions such as obesity [4–7], asthma [8], flu vaccination [9], hypertension [10], and diabetes [11]. Further, nationally aggregated BRFSS data have also been used to estimate the percentage of US adults keeping a firearm at home [12] and those following recommendations regarding physical activity [13] and muscle strengthening [14]. This list is not intended to be comprehensive. Khalil and Crawford [15] identified 1,387 articles using BRFSS data from 1984 through 2012, and noted that in the last 10 years, publications focused on national data were most frequent.

**Table 1:** Respondent totals by survey

Name of survey	Most recent year available	Number
BRFSS	2014	464,664
NHIS	2014	112,053
NHANES^a^	2011–2012	9,756
NSDUH	2014	67,901

^a^Number of respondents to the NHANES household questionnaire

The development of national weights--as well as a methodology for computing the associated variance estimates--is warranted, given the variation in sampling at the state level, and the use of aggregated BRFSS data by many authors. The general methodology presented here, to apply a national weight to the state BRFSS samples, was first developed more than a decade ago [16] based on traditional methods for stratified random sampling [17]. The new methods are more powerful as they draw upon the common sampling and weighting (raking) methodology now used by all states. This article also provides a comparison of the methods based on the variances induced by unequal weighting as well as the bias reduction induced by raking at the national level, and recommends a preferred method.

Combining the BRFSS state-level survey data into a national data set is a necessary initiative for the following reasons:

- ■ The system’s surveys use the same basic sampling methodology across states;
- ■ These surveys produce state-level weights using the same basic methodology;
- ■ The surveys use the same core questionnaire across states;
- ■ BRFSS currently provides technical assistance to data users on a number of other analyses.

In 2011, the adoption of a raking methodology for post-stratification weight adjustments across all states strengthened the foundation for the development of a statistically valid national weighting methodology. A general overview of raking and its applications in combination with trimming is provided in Iachan [18] and in Battaglia, Frankel and Link [19]; the method adopted by the BRFSS is described in CDC’s documentation [1].

## Methods

This paper examines alternative approaches for generating national weights. The data file used in these analyses was the 2013 BRFSS public-use data file. These approaches all begin with the state-level weights now computed in the BRFSS system. The baseline method for our comparisons is a simple method that concatenates the data with the current state-level weights. Among the several limitations of this simple method, perhaps the most important is that the weighted distribution across key demographics does not necessarily match known national demographic distributions. To the extent that survey outcomes are associated with these demographic characteristics, matching the national distributions may reduce bias in estimates of these outcomes at the national level.

The current BRFSS state-level weighting methodology includes a raking process, an iterative form of post-stratification that ensures that weights sum to known population totals for key demographics in each state. Some (but not all) of the new methods developed for national weighting involve an additional layer for the raking that adds the state as a margin. This step ensures that using the national weights at the state level will reproduce the usual state estimate, for every state and every estimate.

An assessment of the weights considers estimated bias and variances, as well as the mean squared error (MSE) for key health risk indicators. While a direct measure of bias is available for key demographic variables, an indirect or estimated bias is necessary for other variables including health outcomes. We compare the national estimates with a benchmark provided by the National Health Interview Survey (NHIS) data for comparable health indicators. The NHIS was chosen as a standard because it provides both the largest sample and a questionnaire that is similar to the BRFSS. NHIS also provides summary annual estimates [20] produced using data fielded during the same time period as the BRFSS. The NHIS is itself a survey and therefore is subject to measurement error within its estimation. Despite the known internal variance within estimates derived from the NHIS, its use as a validation tool is widely accepted. A number of studies have used NHIS to validate estimates from the BRFSS in the past [21–24]. We developed a range of weighting methods that may improve upon the method that aggregates the BRFSS using state-level weights to form a national data set.

### State weights

The state-level weights are the foundations on which the national weights will be computed in the second part of the methods. The weights start from design weights—also known as base weights or sampling weights—computed as the reciprocal of the probabilities of selection. States choose to stratify samples by geographic regions. The states make use of disproportional stratified sampling for fielding efficiency, and the design weights reflect these differential selection probabilities. The design weights also include a correction for the use of overlapping dual landline and cell phone frames. Finally, the weights are raked [19], iteratively fitted to population distributions used as margins shown in Table 2. The BRFSS uses both the American Community Survey (ACS) and Nielsen Claritas for control totals to weight data at the state and sub-state regional level, with the exception of phone usage, which is taken from the National Center for Health Statistics (NCHS) [1].

**Table 2:** Current state-level raking margins^a^

Margin	Categories
1: Sex by Age	Male and Female by Age (18–24; 25–34; 35–44; 45–54; 55–64; 65–74; 75+)
2: Race/Ethnicity	Hispanic, Non-Hispanic White, Non-Hispanic African American, Non-Hispanic Other (includes Asian, American Indian/Alaska Native, Pacific Islander, and Other)
3: Education	Less than HS; HS Grad; Some College; College Grad
4: Marital Status	Married; Never married/member of unmarried couple; Divorced/widowed/separated.
5: Home Ownership	Own; Rent/Other
6: Sex by Race/Ethnicity	Male; Female by Hispanic, Non-Hispanic White, Non-Hispanic African American, Non-Hispanic Other (includes Asian, American Indian/Alaska Native, Pacific Islander, and Other)
7: Race/Ethnicity by Age	Hispanic, Non-Hispanic White, Non-Hispanic African American, Non-Hispanic Other (includes Asian, American Indian/Alaska Native, Pacific Islander, and Other) by Age (18–34; 35–54; 55+)
8: Phone Usage	Cell Only; Landline Only; Dual Usage

^a^Categories may be collapsed in BRFSS raking depending on the size of population subgroups within states

### Variances

As would be expected, there is variability in state-level weights (design weights or sampling weights), which reflects the unequal sampling rates adopted across states. Because the base weights are computed as the reciprocal of sampling probabilities, and for a stratified random sampling design, the probabilities are, in essence, sampling rates in different strata and overall.

Because sample sizes are not proportional to state population sizes, the sampling rates are much larger in the smaller states than in the larger states, as illustrated in Table 3. The table shows that the sampling rate is .05% or less in large states, such as California, New York and Texas; by contrast, the sampling rate is higher than 1.0% for small states such as Nebraska, Montana, South Dakota, and Wyoming.

**Table 3:** Design effect due to the unequal sampling design effect (2013)

	Number	Adult population size	Sampling rate	Design effect	Margin of error	Expected margin of error
Nationwide	483,865	237,659,116	0.20%	4.45	0.14%	0.30%
Alabama	6,503	3,675,910	0.25%	2.19	1.22%	1.80%
Alaska	4,578	532,446	0.82%	2.10	1.45%	2.10%
Arizona	4,252	4,858,658	0.15%	3.21	1.50%	2.69%
Arkansas	5,268	2,223,405	0.23%	2.14	1.35%	1.97%
California	11,518	28,416,963	0.05%	1.96	0.91%	1.28%
Colorado	13,649	3,891,264	0.31%	1.76	0.84%	1.11%
Connecticut	7,710	2,779,516	0.32%	2.20	1.12%	1.65%
Delaware	5,206	703,509	0.74%	1.92	1.36%	1.88%
DC	4,931	514,080	0.74%	2.76	1.40%	2.32%
Florida	34,186	15,084,361	0.05%	5.16^a^	0.53%	1.20%
Georgia	8,138	7,322,131	0.08%	1.96	1.09%	1.52%
Hawaii	7,858	1,071,394	0.71%	2.18	1.11%	1.63%
Idaho	5,630	1,156,346	0.51%	2.18	1.31%	1.93%
Illinois	5,608	9,762,138	0.06%	2.12	1.31%	1.90%
Indiana	10,338	4,917,721	0.18%	1.80	0.96%	1.29%
Iowa	8,157	2,337,531	0.31%	1.82	1.09%	1.46%
Kansas	23,282	2,143,345	0.55%	1.60	0.64%	0.81%
Kentucky	11,013	3,340,703	0.34%	2.42	0.93%	1.45%
Louisiana	5,251	3,452,150	0.26%	2.64	1.35%	2.20%
Maine	8,097	1,059,215	0.94%	1.79	1.09%	1.46%
Maryland	13,011	4,485,506	0.29%	2.51	0.86%	1.36%
Massachusetts	15,071	5,197,008	0.42%	2.56	0.80%	1.28%
Michigan	12,759	7,582,340	0.14%	1.93	0.87%	1.20%
Minnesota	14,340	4,067,360	0.30%	3.43	0.82%	1.51%
Mississippi	7,453	2,228,376	0.35%	2.25	1.14%	1.70%
Missouri	7,118	4,594,138	0.15%	2.29	1.16%	1.76%
Montana	9,693	775,259	1.12%	1.98	1.00%	1.40%
Nebraska	17,139	1,381,509	1.39%	2.78	0.75%	1.25%
Nevada	5,101	2,067,996	0.23%	3.48	1.37%	2.56%
New Hampshire	6,463	1,038,311	0.73%	1.85	1.22%	1.66%
New Jersey	13,386	6,785,166	0.23%	2.29	0.85%	1.28%
New Mexico	9,316	1,555,803	0.56%	2.20	1.02%	1.51%
New York	8,979	15,196,034	0.04%	1.84	1.03%	1.40%
North Carolina	8,860	7,369,782	0.16%	1.90	1.04%	1.43%
North Dakota	7,806	535,913	0.91%	2.08	1.11%	1.60%
Ohio	11,971	8,853,774	0.15%	2.25	0.90%	1.34%
Oklahoma	8,244	2,850,383	0.28%	1.76	1.08%	1.43%
Oregon	5,949	3,006,433	0.18%	1.84	1.27%	1.72%
Pennsylvania	11,429	9,971,001	0.20%	1.83	0.92%	1.24%
Rhode Island	6,531	831,949	0.66%	1.96	1.21%	1.70%
South Carolina	10,717	3,600,525	0.36%	2.10	0.95%	1.37%
South Dakota	6,895	621,017	1.27%	2.89	1.18%	2.00%
Tennessee	5,815	4,909,634	0.14%	2.13	1.29%	1.88%
Texas	10,917	18,714,465	0.05%	2.54	0.94%	1.49%
Utah	12,769	1,934,173	0.64%	1.71	0.87%	1.13%
Vermont	6,392	499,262	1.21%	1.76	1.23%	1.63%
Virginia	8,464	6,244,639	0.12%	1.92	1.07%	1.47%
Washington	11,162	5,234,679	0.29%	1.91	0.93%	1.28%
West Virginia	5,899	1,468,456	0.37%	1.47	1.28%	1.55%
Wisconsin	6,589	4,381,727	0.12%	2.57	1.21%	1.94%
Wyoming	6,454	433,712	1.45%	2.09	1.22%	1.76%

^a^The reason for the high Florida design effect is because they oversampled smaller counties that particular year. They do this every 3 years in order to have direct estimates for each county in the state. This design leads to highly unequal probabilities of selection across counties in the state

Table 3 also presents the design effect (DEFF) due to weighting at the state level, the component of the DEFF due to unequal weighting effects. It gauges the impact of the weight variability on sampling error under two scenarios:

- under simple random sampling, and
- by allowing for the impact of unequal weighting effects.

The measure of sampling error shown in this table is the margin of error, i.e., the half-width of a 95% confidence interval. It is also worth noting that design effects are high for Florida as the state oversampled smaller counties that year, as it does every 3 years.

The national design effect of 4.49, which applies to national estimates produced using the concatenated state-level weights, is substantial. This design effect more than doubles the margin of error on such estimates due to the additional variance introduced by the concatenated or aggregated weights. Reduction of variance using a national weighting method, rather than aggregating the state weights would therefore be preferable.

### Bias and raking

It is reasonable to assume that the use of the aggregated state-level weights may lead to biases at the national level to the extent that for key demographics, as the aggregated weighted distribution does not match the national population distribution. For example, although each state’s population is appropriately weighted, the estimated percentage for Hispanics is 15.5% with the aggregated while a national weighting method would reduce that proportion to 15%, a more accurate representation of national percentages. The demographic biases in the aggregated method, therefore, may have implications for health outcomes that may show variations across demographic groups. To control for this potential bias, the national weights could be raked at the national level using as many of the raking dimensions—among those used at the state level—as possible for convergence and stability. In addition, national raking could use states as an additional margin to preserve the state totals and to reproduce state estimates. We therefore produced a series of reweighting methods using a range of raking margins defined in Table 4, in addition to the state-level margins defined in Table 2. Some of the national raking methods add additional margins to the first eight, starting with the overall state margins and then adding cross-classifications of state with key demographic variables. Each of these reweighting methods start with the original BRFSS design weights and readjusted the raking process at the national level.

**Table 4:** Groups of national raking margins and corresponding weighting methods

Method	Margins	Categories
1	1–8	1: Sex by Age 2: Race/Ethnicity 3: Education 4: Marital Status 5: Home Ownership 6: Sex by Race/Ethnicity 7: Race/Ethnicity by Age 8: Phone Usage
2	1–8 + 9	1: Sex by Age 2: Race/Ethnicity 3: Education 4: Marital Status 5: Home Ownership 6: Sex by Race/Ethnicity 7: Race/Ethnicity by Age 8: Phone Usage 9: State
3	1–8 + state with 3 cross classifications	1: Sex by Age 2: Race/Ethnicity 3: Education 4: Marital Status 5: Home Ownership 6: Sex by Race/Ethnicity 7: Race/Ethnicity by Age 8: Phone Usage 9: State 10: Age by state 11: Sex by state 12: Race/ethnicity by state
4	1–8 with collapsed categories^a^	1: Sex by Age 2: Race/Ethnicity 3: Education 4: Marital Status 5: Home Ownership 6: Sex by Race/Ethnicity (collapsed categories) 7: Race/Ethnicity by Age (collapsed categories) 8: Phone Usage
5	1–8 + state with collapsed categories	1: Sex by Age 2: Race/Ethnicity 3: Education 4: Marital Status 5: Home Ownership 6: Sex by Race/Ethnicity (collapsed categories) 7: Race/Ethnicity by Age (collapsed categories) 8: Phone Usage 9: State
6	1–8 + state with 3 cross classifications with collapsed categories^b^	1: Sex by Age 2: Race/Ethnicity 3: Education 4: Marital Status 5: Home Ownership 6: Sex by Race/Ethnicity 7: Race/Ethnicity by Age 8: Phone Usage 9: State 10: Age by state (collapsed categories) 11: Sex by state 12: Race/ethnicity by state (collapsed categories)

^a^In Methods 4–6, margins 6 and 7 were collapsed to achieve minimum sample sizes of 300 or minimum sample percentages of 5.0%. Race/ethnicity in margin 6 was collapsed to non-Hispanic White and Other for males; non-Hispanic White, non-Hispanic Black, and Other for females. In margin 7, race/ethnicity was collapsed to non-Hispanic White and Other

^b^Margins 10 and 12 were collapsed within region to achieve minimum sample sizes of 250 or minimum sample percentages of 5.0%. The age categories of 18–24 and 25–34 were collapsed together in margin 10 for 16 states. In margin 12, all race/ethnicity categories were collapsed together for two states (Maine and Vermont)

The first reweight uses the original raking margins as described in Table 2, but readjusts to reflect a single national demographic weighting rather than merely aggregating the states’ unequal samples. The second reweight uses the original eight raking margins as well as state (Margin 9). The third reweight includes three classifications (age, sex and race/ethnicity) by state. An additional three reweighting methods are tested in an effort to reduce the overall variability of the weights. These three methods use the same overall raking margins as the first three methods but collapse some demographics (race and age) into larger categories. Some additional collapsing of margins is performed on individual cells to ensure that all cells obtained a minimum sample sizes of 300 or a minimum sample percentage of 5.0%. In Methods 4–6, margins 6 and 7 were collapsed. Race/ethnicity in margin 6 was collapsed to non-Hispanic White and Other for males; non-Hispanic White, non-Hispanic Black, and Other for females. In margin seven, race/ethnicity was collapsed to non-Hispanic White and Other.

In total, six national weighting strategies are tested: Method 1 uses the same margins as the original BRFSS, but weighted at the national level; Method 2 uses the BRFSS margins at the national level and adding state; Method 3 uses the BRFSS margins, and adding state with three additional state cross categories; Method 4 uses the BRFSS margins in collapsed categories; Method 5 uses the BRFSS margins plus state in collapsed categories, and Method 6 uses the BRFSS margins, state and the cross-classifications by state in collapsed categories (see Table 4).

## Results

The methods are compared in terms of the estimated variance and bias of resulting weighted survey estimates. The estimated variances are gauged in two ways. First, in terms of the variability in the weights, we assessed a pure contribution of unequal weighting to the design effects and survey variances. Second, using a more empirical approach, we looked at the estimated variances for a number of key health indicators. The indicators are for current smoking, diabetes, arthritis, asthma, stroke, lack of insurance, obesity, and HIV testing. Finally a single indicator, diabetes, is examined by demographic subgroup to examine whether some of the methods may perform better for subgroup estimates.

We begin comparing the biases in the different weighted estimates using the aggregated, traditional method and the six new national weighting methods. The biases are estimated by comparing the weighted estimates with a benchmark available from the National Health Interview Survey (NHIS), specifically, from Tables of Summary Health Statistics for 2013 [4].

Weighted prevalence estimates for a number of key health indicators are presented in Table 5 using the aggregated, traditional method and the six new national weighting methods together with the NHIS annual summary estimates [20] for the same or similar indicators. The NHIS estimates also permit the computation of a reduction in Mean Squared Error (MSE), estimated as the variance plus the square of the bias (the absolute difference between the weighted estimate and the benchmark NHIS estimate (MSE = SE^2^ + [Percent – Percent NHIS]^2^)).

**Table 5:** Comparison of prevalence estimates by Method and NHIS Benchmark^a^

Weighting method	Current smoker	Ever told had diabetes	Ever told had arthritis	Ever told had asthma	Obesity	Ever told had stroke	Uninsured (Among 18–64)	Ever had hiv test	Average MSE
Aggregated Weights	18.23%	10.22%	25.02%	14.03%	28.29%	2.93%	17.46%	37.63%	0.015%
	SE: 0.12	SE: 0.09	SE: 0.12	SE: 0.10	SE: 0.14	SE: 0.04	SE: 0.13	SE: 0.16	
	MSE: 0.002	MSE: 0.008	MSE: 0.053	MSE: 0.049	MSE: 0.001	MSE: 0.000	MSE: 0.003	MSE: 0.001	
Method 1	18.57%	10.22%	25.15%	13.98%	28.96%	2.99%	17.93%	37.06%	0.017%
	SE: 0.11%	SE: 0.08	SE: 0.10	SE: 0.10	SE: 0.13	SE: 0.04	SE: 0.12	SE: 0.14	
	MSE: 0.006	MSE: 0.008	MSE: 0.059	MSE: 0.047	MSE: 0.001	MSE: 0.001	MSE: 0.010	MSE: 0.001	
Method 2	18.30%	10.19%	25.04%	14.10%	28.62%	2.97%	17.64%	37.69%	0.015%
	SE: 0.11	SE: 0.08	SE: 0.11	SE: 0.10	SE: 0.13	SE: 0.04	SE: 0.12	SE: 0.15	
	MSE: 0.003	MSE: 0.007	MSE: 0.053	MSE: 0.052	MSE: 0.000	MSE: 0.000	MSE: 0.005	MSE: 0.002	
Method 3	18.34%	10.19%	25.04%	14.11%	28.63%	2.97%	17.67%	37.76%	0.016%
	SE: 0.11	SE: 0.08	SE: 0.11	SE: 0.10	SE: 0.13	SE: 0.04	SE: 0.12	SE: 0.15	
	MSE: 0.003	MSE: 0.007	MSE: 0.053	MSE: 0.053	MSE: 0.000	MSE: 0.000	MSE: 0.006	MSE: 0.002	
Method 4	18.56%	10.23%	25.16%	13.96%	28.97%	2.99%	17.93%	37.02%	0.016%
	SE: 0.11	SE: 0.08	SE: 0.10	SE: 0.10	SE: 0.13	SE: 0.04	SE: 0.12	SE: 0.14	
	MSE: 0.006	MSE: 0.008	MSE: 0.059	MSE: 0.046	MSE: 0.001	MSE: 0.001	MSE: 0.010	MSE: 0.001	
Method 5	18.29%	10.20%	25.05%	14.08%	28.63%	2.97%	17.63%	37.65%	0.015%
	SE: 0.11	SE: 0.08	SE: 0.11	SE: 0.10	SE: 0.13	SE: 0.04	SE: 0.12	SE: 0.15	
	MSE: 0.002	MSE: 0.007	MSE: 0.054	MSE: 0.051	MSE: 0.000	MSE: 0.000	MSE: 0.005	MSE: 0.001	
Method 6	18.33%	10.20%	25.04%	14.09%	28.64%	2.96%	17.65%	37.72%	0.015%
	SE: 0.11	SE: 0.08	SE: 0.11	SE: 0.10	SE: 0.13	SE: 0.04	SE: 0.12	SE: 0.15	
	MSE: 0.003	MSE: 0.007	MSE: 0.053	MSE: 0.052	MSE: 0.000	MSE: 0.000	MSE: 0.005	MSE: 0.002	
NHIS estimate	17.8%	9.5%	22.7%	11.8%	28.6%	2.8%	16.7%	37.3%	
	SE: 0.30	SE: 0.20	SE: 0.32	SE: 0.23	SE:.36	SE: 0.11	SE: 0.25	SE: 0.41	

^a^Although both BRFSS and NHIS collect information on these outcomes, there are minor differences in question wording between the two surveys, as well as differences in the mode of administration

There are little to no differences in the MSE reduction among the methods for the responses to the questions on stroke and insurance, but more discernable differences in the question on whether respondents had ever had asthma. While each method reduces the MSE by .012 to .013, making it difficult to ascertain differences between them, methods 4 and 2 perform better than others when estimates are compared against the NHIS benchmark.

Since health conditions vary by demographic characteristics, subgroups of respondents were examined for differences on responses to the diabetes question (see Table 6). Diabetes was selected, since it is a condition that varies by demographic group. Table 6 shows that for Hispanic group estimates, the MSE is lowest for Method 4.

**Table 6:** Comparison of weighting methods and NHIS diagnosed diabetes prevalence estimates by respondent demographic characteristics

	Male	Female	Age 18–44	Age 45–64	Age 65–74	Age 75+	White	Black	Hisp.	Less Than HS	HS only	Some college
Aggregated Weights	10.42%	10.04%	2.88%	13.69%	22.73%	21.62%	9.58%	14.13%	10.63%	15.82%	11.25%	8.23%
	SE: 0.13	SE: 0.12	SE: 0.08	SE: 0.18	SE: 0.32	SE: 0.37	SE: 0.09	SE: 0.32	SE: 0.32	SE:0.35	SE: 0.16	SE: 0.10
	MSE: 0.005	MSE: 0.011	MSE: 0.001	MSE: 0.019	MSE: 0.029	MSE: 0.007	MSE: 0.003	MSE: 0.061	MSE: 0.004	MSE:0.031	MSE: 0.003	MSE: 0.003
Method 1	10.39%	10.07%	2.99%	13.75%	22.98%	21.61%	9.58%	13.54%	10.81%	15.80%	11.26%	8.23%
	SE: 0.12	SE: 0.10	SE: 0.08	SE: 0.16	SE: 0.27	SE: 0.31	SE: 0.08	SE: 0.35	SE: 0.30	SE: 0.32	SE: 0.14	SE: 0.08
	MSE: 0.004	MSE: 0.012	MSE: 0.001	MSE: 0.021	MSE: 0.038	MSE: 0.006	MSE: 0.003	MSE: 0.035	MSE: 0.006	MSE: 0.030	MSE: 0.003	MSE: 0.003
Method 2	10.38%	10.02%	2.88%	13.78%	22.84%	21.50%	9.55%	13.46%	10.88%	15.90%	11.20%	8.18%
	SE: 0.12	SE: 0.10	SE: 0.08	SE: 0.16	SE: 0.27	SE: 0.33	SE: 0.09	SE: 0.28	SE: 0.31	SE: 0.33	SE: 0.14	SE: 0.09
	MSE: 0.004	MSE: 0.011	MSE: 0.001	MSE: 0.022	MSE: 0.033	MSE: 0.005	MSE: 0.002	MSE: 0.032	MSE: 0.007	MSE: 0.033	MSE: 0.003	MSE: 0.003
Method 3	10.39%	10.00%	2.89%	13.81%	22.80%	21.37%	9.57%	13.40%	10.95%	15.89%	11.17%	8.19%
	SE: 0.12	SE: 0.11	SE: 0.08	SE: 0.17	SE: 0.29	SE: 0.33	SE: 0.09	SE: 0.29	SE: 0.32	SE: 0.34	SE: 0.14	SE: 0.09
	MSE: 0.004	MSE: 0.011	MSE: 0.001	MSE: 0.023	MSE: 0.032	MSE: 0.003	MSE: 0.003	MSE: 0.030	MSE: 0.009	MSE: 0.033	MSE: 0.002	MSE: 0.003
Method 4	10.37%	10.09%	2.92%	13.73%	23.01%	21.67%	9.53%	14.40%	10.44%	15.72%	11.30%	8.24%
	SE: 0.12	SE: 0.10	SE: 0.08	SE: 0.15	SE: 0.26	SE: 0.32	SE: 0.08	SE: 0.28	SE: 0.29	SE: 0.32	SE: 0.14	SE: 0.08
	MSE: 0.004	MSE: 0.012	MSE: 0.001	MSE: 0.021	MSE: 0.039	MSE: 0.007	MSE: 0.002	MSE: 0.074	MSE: 0.002	MSE: 0.027	MSE: 0.004	MSE: 0.003
Method 5	10.36%	10.05%	2.89%	13.76%	22.87%	21.57%	9.52%	14.33%	10.60%	15.85%	11.24%	8.19%
	SE: 0.12	SE: 0.10	SE: 0.08	SE: 0.16	SE: 0.27	SE: 0.33	SE: 0.08	SE: 0.29	SE: 0.30	SE: 0.33	SE: 0.14	SE: 0.09
	MSE: 0.004	MSE: 0.011	MSE: 0.001	MSE: 0.022	MSE: 0.034	MSE: 0.006	MSE: 0.002	MSE: 0.070	MSE: 0.004	MSE: 0.031	MSE: 0.003	MSE: 0.003
Method 6	10.38%	10.03%	2.90%	13.80%	22.82%	21.41%	9.57%	14.02%	10.97%	15.91%	11.20%	8.19%
	SE: 0.12	SE: 0.11	SE: 0.08	SE: 0.17	SE: 0.28	SE: 0.34	SE: 0.09	SE: 0.30	SE: 0.31	SE: 0.34	SE: 0.14	SE: 0.09
	MSE: 0.004	MSE: 0.011	MSE: 0.001	MSE: 0.023	MSE: 0.033	MSE: 0.004	MSE: 0.003	MSE: 0.055	MSE: 0.009	MSE: 0.034	MSE: 0.003	MSE: 0.003
NHIS Estimate	9.9%	9.1%	2.7%	12.5%	21.6%	21.6%	9.2%	11.9%	9.7%	16.4%	12.6%	10.7%
	SE: .30	SE: .26	SE: .16	SE: .41	SE: .82	SE: .93	SE: .23	SE: .56	SE: .47	SE: .67	SE:.50	SE: .43

The BRFSS calculates a design weight for each respondent based on the probability of selection. This weight takes into account the number of adults and telephones within each household as well as the size of the sample drawn within each state and substate region [1]. Table 7 presents the variability in the weights as measured by the design effect (DEFF) due to unequal weighting for each method. It also shows the margin of error (half-width for the 95% confidence interval) for each method. The table suggests a slight superiority for the two methods using 8 marginal classes – that is, a reduction in the variance of the national weights, which translates into more precise national estimates. Table 7 also indicates that Method 4 has the lowest design effect of 3.92, as well as a comparatively low coefficient of variation at 1.71. We stress that this analysis is confined to the DEFF component due to unequal weighting effects, and therefore, do not reflect the variance gains induced by stratification (e.g., by states). The stratification effects, or gains, are the same across all the national weighting methods. Incorporating these gains in the variance estimation process is also an important element of the national weighting estimation strategy developed in this research.

**Table 7:** Weight variability by National Weighting Method

National weighting method	CV^a^	Design effect	Expected margin of error
Aggregated State Weights	1.86	4.45	0.30%
Method 1: 8 Margins	1.71	3.93	0.28%
Method 2: 9 Margins	1.80	4.26	0.29%
Method 3: 12 Margins	1.79	4.22	0.29%
Method 4: 8 Collapsed Margins	1.71	3.92	0.28%
Method 5: 9 Collapsed Margins	1.80	4.24	0.29%
Method 6: 12 Collapsed Margins	1.79	4.21	0.29%

^a^Coefficient of variation

**Fig. 1:**
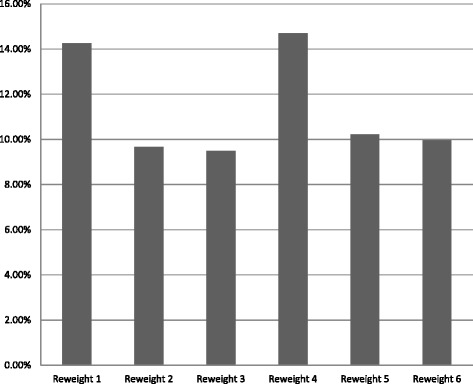
Average variance reduction relative to aggregated weights

Figure 1 shows the relative reduction in variance of the weights, compared with the aggregated (baseline) approach. This measure of relative reduction is based on the average variance of the key estimates considered in this empirical investigation. Specifically, the relative reduction in variance is computed as (*V*_*i*_ − *V*_0_)/*V*_0_, where *V*_*i*_ is the average variance under the weighting method *i* and *V*_0_ is the average variance under the aggregated method. The figure shows that the largest reductions in average variance are achieved by the two methods with eight margins—i.e., Method 1 (without collapsing) and Method 4 (with collapsing), each reducing the variance in the weights by more than 14 %.

When demographic characteristics are taken into account, some differences are noted among the methods in that there is more variance. Of the national weighting methods, Method 4 performs better in terms of the NHIS benchmark, producing estimates closest to the NHIS benchmarks in five of the 12 cases. In addition, Method 4 reduces the MSE by a greater proportion than the other methods.

Thus Method 4 illustrates superiority over the other methods in terms of reduction in design effect and variance, and comes closer to matching national estimates from an outside source.

## Discussion

The increased uniformity of BRFSS sampling and weighting methods across states since 2011 makes the aggregation more efficient than in earlier investigations, starting in the late 1990s and early 2000s [16]. At that time, the variation in the sampling and weighting methodologies across states created additional challenges.

One additional motivation for the BRFSS data weighting methods to national population totals is the fact that there are unequal selection probabilities among the state samples. It is clear that the design effect at the national level is high and that the methods proposed decrease the variance of the weights (as shown in Fig. 1).

For the limited set of estimates compared against the NHIS national estimates, the aggregated method of weighting produced estimates that were not statistically different than those of other weighting methods tested (based on chi-square tests or t-tests of significance). Data users who conduct other analyses using additional variables and methods, however, have no prior knowledge of the degree to which the use of national weights will reduce bias in their outcomes. What is known is that the national weighting methods will lead to reductions in variance due to unequal weighting effects; in addition, the new methods will also account for the demographic biases built into the multiple sampling designs adopted by the states. The incentive for the adoption of national weighting comes from the reduction in the variance in the weights and improvement in demographic representation at the national level. Such improvements are the core of the argument in favor of national weights.

While the reduction of MSE overall is small among weight methods tested, Method 4 is superior to the other weighting methods in terms of lower variance in weights (see Fig. 1). It also has a lower overall design effect than other methods (see Table 6) and uses collapsed margins, making it somewhat more efficient to produce. When we compared prevalence estimates against those of the NHIS benchmark, we found that it performed better than other national weighting strategies. Method 4 is similar to the weight method used for individual states in that the margins are the same, but adjustments to the control totals are made to account for the national population, rather than aggregating from the state weighted totals. It is also worth noting that our updated recommendations, using 2013 as well as 2012 BRFSS data and focused more on variances, are not exactly the same as the more mixed picture depicted in national conferences (e.g., [25]).^1^ The previous work was more focused on bias reduction where the methods seem equivalently effective at the national level. That work was also focused on a smaller subset of health indicators and older BRFSS data (2012 versus 2013).

## Conclusions

The methodology described in this paper provides national weights for the state-based BRFSS. Data users who aggregate data from all states would benefit from the use of these new national weights. Persons using data from only a few states would find that the weights associated with state level populations would be better suited to their analyses; an analysis that used data from a BRFSS module administered to residents in only a few states should use state-level weights rather than a national weight. Users should always take care to include complex sample designs in any and all analyses, which included BRFSS data, as they are both collected using stratified and weighted designs. Technical documentation indicate the weighting variables for data users on the BRFSS website [2].

Unlike the usual aggregated approach, the new methods lead to weighted distributions that reproduce national population distributions for all key demographic groupings. To the extent that survey outcomes are associated with these demographic characteristics, matching the national distributions will reduce bias in estimates of these outcomes at the national level.

## Abbreviations

ACS: American Community Survey
BRFSS: Behavioral risk factor surveillance system
CDC: Centers for disease control and prevention
CV: Coefficient of variation
DEFF: Design effect
MSE: Mean squared error
NCHS: National Center for Health Statistics
NHIS: National Health Interview Survey
SE: Standard error

## Footnotes

1 The paper presented at the 2014 Joint Statistical Meetings available at https://www.amstat.org/sections/srms/proceedings/y2014/Files/313745_91661.pdf.
